# Aspirin protects against genotoxicity by promoting genome repair

**DOI:** 10.1038/s41422-023-00783-6

**Published:** 2023-03-01

**Authors:** Hui Jiang, Patrycja Swacha, Kyaw Min Aung, Nelson O Gekara

**Affiliations:** 1 Institute of Medical Microbiology and Hygiene, Medical Center - University of Freiburg, Faculty of Medicine, Freiburg, Germany; 2 Department of Molecular Biosciences, The Wenner-Gren Institute, Stockholm University, Stockholm, Sweden

**Keywords:** Double-strand DNA breaks, Toll-like receptors

Dear Editor,

Radiation sickness is a major health concern.^1^ The quest for radiation countermeasures started in the wake of the devastation witnessed following the nuclear detonations during the Second World War and has continued through the subsequent radiological accidents around the world. A radioprotector is also required for prophylactic use by staff working at radiation sources, pilots, and astronauts at high risk of space radiation, or patients undertaking lengthy radiological procedures. Despite decades of research, a safe, efficient, and cost-effective radioprotector is yet to be unveiled.

Acetylsalicylic acid (aspirin) is the oldest drug in the history of medicine. It has been used for over 4000 years for the treatment of pain, inflammation, fever, and more recently for cardiovascular prophylaxis^2^ and cancer prevention.^3^

Bone marrow failure is the primary cause of mortality following irradiation. Hence, protecting the bone marrow is a primary goal in the development of radiation countermeasures. Inflammation is a key outcome and driver of irradiation-induced tissue injury.^4^ Given its anti-inflammatory effects, we inquired whether aspirin could protect against radiation. When inoculated into mice, aspirin protected against irradiation-induced bone marrow ablation (Fig. 1a; Supplementary information, Fig. S1a–d) and suppressed the induction of inflammatory genes including *Ifnb1, Mx1* and *Tnfa* in vivo and in bone marrow-derived monocytes (BMDMos) (Supplementary information, Figs. S1e–g and S2a–c).

**Fig. 1:**
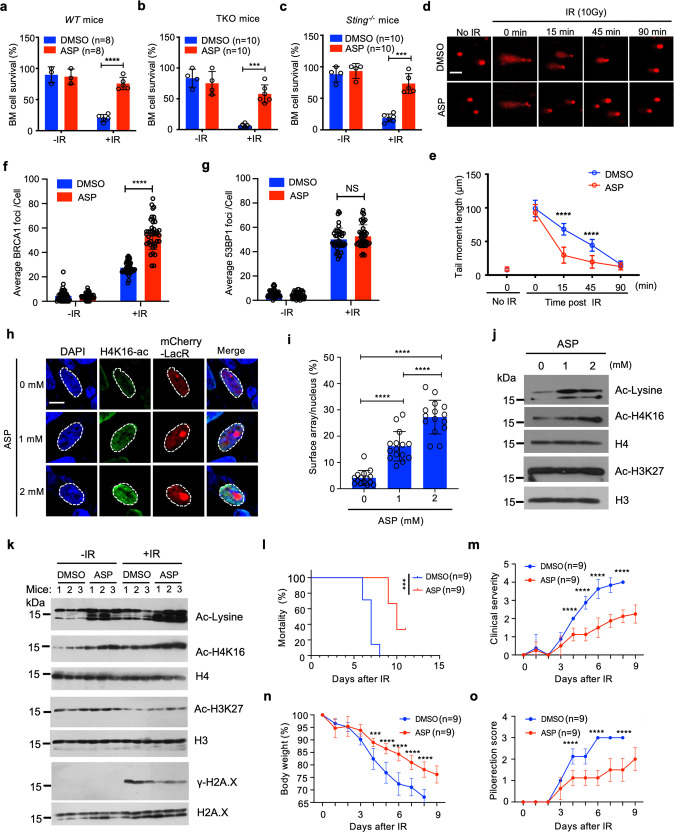
Aspirin protects against radiation sickness by promoting HR-DNA repair. **a–c** Survival of bone marrow (BM) cells 10 h post irradiation (9 Gy) of WT mice (**a**), *Myd88*^*−/−*^*Trif*^*−/−*^*Mavs*^*−/−*^ (TKO) mice (**b**) and *Sting*^*−/−*^ mice (**c**) pretreated with aspirin or DMSO. Data are presented as means ± SD. One-way ANOVA test, ^∗∗∗^*P* < 0.001, ^∗∗∗∗^*P* < 0.0001. **d**, **e** Aspirin promotes DNA repair. Images (**d**) and corresponding quantification (**e**) of the comet tails of BMDMos pre-treated with aspirin or DMSO, irradiated (9 Gy on ice), then incubated at 37 °C to allow DNA repair. Scale bar, 50 μm. **f**, **g** BRCA1 foci (**f**) and 53BP1 foci (**g**) per nucleus 1 h after irradiation (9 Gy) of HEK293 cells pre-treated with DMSO or aspirin. Data are presented as means ± SEM, *n* = 30. One‐way ANOVA test, NS, *P* > 0.5, ****P < 0.0001. **h**, **i** Aspirin induces chromatin decompaction. Confocal images of mCherry-LacR in aspirin-treated AO3 cells (**h**) and corresponding quantification of the relative array size (surface of the mcherry-LacR array/surface (**i**). Data are presented as means ± SEM. Two-tailed Student’s *t*-test, ****P < 0.0001. Scale bar = 10 μm. **j** Histone acetylation marks in chromatin isolated from aspirin-treated AO3 cells. **k** Aspirin induces Ac-H4K16 in vivo and is associated with reduced markers of DNA damage. Histone acetylation and γ-H2A.X in chromatin from bone marrow cells of mice that were treated with aspirin (or not) followed by irradiation. **l**–**o** Aspirin protects against radiation sickness and extends survival. Mortality (**l**), clinical severity (**m**), weight loss (**n**) and piloerection score (**o**) in aspirin-treated and untreated mice following γ-irradiation (18 Gy). Differences in morbidity (**l**) were analyzed by log-rank test, ***P < 0.001. Graphs in **m**–**o** were shown as means ± SEM. One-way ANOVA test, ^∗∗∗^*P* < 0.001.

Pattern recognition receptors (PRRs) including Toll-like receptors (TLRs), RIG-I-like receptors (RLRs), and cytosolic DNA sensors (CDS) are central to the initiation of inflammation and cell death.^5^ PRRs signal via key adaptors including MYD88 and TRIF (for TLRs), MAVS (for RLRs), and STING (for CDS) (Supplementary information, Fig. S3a). To assess the impact of aspirin on PRR pathways, we stimulated BMDMos with specific agonists for PRRs including TLRs (TLR2: Pam3CSK4, TLR3: Poly(I:C)), RIG-I (Poly(I:C) transfection), cGAS-STING (poly(dA:dT) or cGAMP transfection) and AIM2 inflammasome (poly(dA:dT)). We found that aspirin inhibited inflammatory gene induction via all these PRRs (Supplementary information, Fig. S3b–f) but not the AIM2 inflammasome (Supplementary information, Fig. S4a–c). To assess if radioprotection by aspirin was due to suppression of PRR-driven inflammation, we compared wild-type (WT) mice with those defective in PRR signaling. Similar to WT (Fig. 1a; Supplementary information, Fig. S1a–d), aspirin protected the bone marrows of triple knockout (TKO) mice that are defective in both TLR and RLR pathways (*Myd88*^*−/−*^*Trif*^*−/−*^*Mavs*^*−/−*^) (Fig. 1b; Supplementary information, Fig. S5a–d), or those defective in cytosolic DNA sensing (*Sting*^*−/−*^) against irradiation (Fig. 1c; Supplementary information, Fig. S6a–d), and suppressed inflammatory gene expression (Supplementary information, Figs. S5e–g and S6e–g). This implied that observed bone marrow suppression was independent of PRR-driven inflammation and that radioprotection by aspirin was uncoupled from its anti-inflammatory effects.

Double-stranded DNA breaks (DSBs) are the most deleterious outcomes of irradiation. Micronuclei are key aftereffects of DSBs.^6^ HEK293 cells are defective in PRR signaling and lack prostaglandin-endoperoxide synthases (COX1 and COX2) — also key mediators of inflammation and pain, and the best-known targets of aspirin.^7,8^ Aspirin suppressed irradiation-induced micronuclei generation in HEK293 cells (Supplementary information, Fig. S7a, b), indicating that such effect was independent of its anti-inflammatory activity. When irradiated on ice (to prevent spontaneous repair), then transferred from ice to 37 °C to allow DNA repair to occur, aspirin pre-treated cells repaired DSBs faster (Fig. 1d, e). Aspirin also accelerated the repair of DSBs induced by the anti-cancer drug doxorubicin (Supplementary information, Fig. S8a, b).

DSB repair occurs via homologous recombination (HR) and Non-Homologous End Joining (NHEJ). Results from GFP-based reporter systems revealed that aspirin promotes the HR but not the NHEJ (Supplementary information, Fig. S9a–e). BRCA1 and 53BP1 are key checkpoint proteins for HR and NHEJ repair, respectively. Aspirin enhanced recruitment of BRCA1 but not the NHEJ repair protein 53BP1 to DNA damage sites (Fig. 1f, g; Supplementary information, Fig. S10). Accordingly, deletion of BRCA1 significantly blunted acceleration of DSB repair by aspirin (Supplementary information, Fig. S11). In contrast, ablation of 53BP1 (Supplementary information, Fig. S12) or inhibition of the NHEJ kinase DNA-PKc did not (Supplementary information, Fig. S13).

Chromatin decompaction is essential for the recruitment of DNA repair machinery to damage sites.^9^ The N-terminal tail of histone H4 is central for inter-nucleosome interaction (Supplementary information, Fig. S15a). Acetylation of histone H4 at lysine K16 (Ac-H4K16) is vital for decreasing the nucleosome–nucleosome stacking and chromatin folding, to permit the recruitment of repair proteins.^10^ Ac-H4K16 also supports the preferential recruitment of BRCA1 over 53BP1 to damage sites, thereby tipping the balance towards HR. Aspirin-treated cells exhibited elevated Ac-H4K16 and recruitment of BRCA1 but not 53BP1 to DNA damage sites (Fig. 1h, I; Supplementary information, Fig. S10a–d). Consistent with direct donation of acetyl groups to targets, aspirin increased Ac-H4K16 in cells treated with the histone acetyltransferase inhibitor (Supplementary information, Fig. S14a, b) or when incubated directly with chromatin isolates (Supplementary information, Fig. S14c). In contrast, acetylated H3K27 was already high at steady state and remained largely unaltered following aspirin (Fig. 1j). Conceivably, given its location at the N-terminal tail of histone H4 where it functions as the first contact point anchoring the H4 tail on the adjacent nucleosome (Supplementary information, Fig S15a), H4K16 is likely more accessible for direct acetylation by aspirin.

To examine whether aspirin modulates chromatin compaction, we employed the AO3 cells containing genomic insertions of multiple copies of the *Escherichia coli* (*E. coli*) lactose operon (LacO) sequence within a heterochromatic region. Upon chromatin decompaction, this region expands, and this can be visualized by expressing fluorescent (mCherry)-tagged *E. coli* lactose repressor protein (LacR) (Supplementary information, Fig. S15b). Aspirin increased Ac-H4K16 and expanded the LacO array (Fig. 1h–j).

To interrogate further, we isolated chromatin from bone marrow cells of mice. Consistent with the ability to accelerate the resolution of DNA breaks in vivo, aspirin-treated mice had elevated Ac-H4K16 and upon irradiation, exhibited decreased levels of the γ-H2A — a marker of DNA damage. When monitored further, aspirin-treated mice had a prolonged survival and exhibited less severe irradiation symptoms (Fig. 1k–o).

The therapeutic effects of aspirin are assumed to be due to its ability to suppress inflammation. Mechanistically, aspirin was originally reported to achieve this via the inhibition of COX1 and COX2.^7,8^ Here we show that while aspirin also suppresses inflammation by blocking multiple PRR pathways, outside its anti-inflammatory effects, aspirin is a potent amplifier of HR-mediated DNA repair. Our data support a model whereby by acetylating the H4K16, aspirin enhances chromatin de-condensation and thereby enhances the recruitment of HR – repair factors to damage sites.

DSBs are deleterious to health. In addition to their potential to trigger cell death, if unrepaired or mis-repaired, DSBs can cause chromosome deletions and translocations leading to long-term deleterious effects, including cancer and hereditary disorders. DSBs are also the cause of inflammation^4^ — a key driver of the radio/chemotherapy-induced tissue injuries such as fibrosis that significantly impact life quality of survivors. In addition to accelerating DSB repair, aspirin could also possibly indirectly contribute to genome protection by suppressing inflammation-induced DNA damage. Thus, the abilities of aspirin to inhibit inflammatory pathways and promote the repair of DSBs via HR underscore its potential in the management of inflammatory and genome instability-driven health afflictions. Further, the discovery that aspirin is a modulator of chromatin structure and repair offers a new mechanism that may explain some of its many acclaimed health benefits including cancer prevention.^3^

## Supplementary information

Supplementary Information
