# Supplementary material for: Aspirin protects against genotoxicity by promoting genome repair
Source: Cell Res. 2023 Mar 1;33(4):325–7. doi: 10.1038/s41422-023-00783-6 (PMC10066308; doi:10.1038/s41422-023-00783-6)
Supplement: Supplementary file 1 — Supplementary Information [file 41422_2023_783_MOESM1_ESM.pdf]

Supplementary Information

Methods and Materials

Mice

All mice in this study were on C57BL/6J background. *Ticam1*$^{-/-}$ (C57BL/6J-Ticam1Lps2/J, stock #005037) and *Sting*$^{-/-}$(C57BL/6J- Tmem173gt/J, stock #017537) [1] mice were from Jackson Laboratory. *Myd88*$^{-/-}$[2] and *Mavs*$^{-/-}$(*Ips1*$^{-/-}$)[3] mice were from S. Akira´s laboratory, Osaka University, Japan. *Myd88*$^{-/-}$, *Trif*$^{-/-}$, *Mavs*$^{-/-}$ mice were crossed with each other to generate the *Myd88*$^{-/-}$ *Trif*$^{-/-}$ *Mavs*$^{-/-}$ mice[4,5]. Mice were bred in specific pathogen-free animal facility at Umeå center for comparative Biology (UCCB). Experiments were carried out according to the guidelines set out by the Umeå Regional Animal Ethic Committee (Umeå Regionala Djurförsöksetiska Nämnd), Approval no. A25-19.

Aspirin treatment and irradiation of mice

Mice were given a daily intraperitoneal (i.p.) injection of DMSO (control) or aspirin (50 mg/kg =0.277mM) for 7 days. To assess the effect of irradiation on the bone marrow, mice were placed in a Gammacell 40 irradiator (MDS Nordion) with a 137 Cs gamma-ray source and given total dose of 9 Gy. 10 hours later they were sacrificed and bone marrow cells were isolated, counted and analyzed by flow cytometry for the following populations: hematopoietic stem cells (HSCs; c-Kit$^+$Sca-1$^+$), B cells (B220$^+$) and neutrophils (Gr1$^+$Ly6G$^+$. The total bone marrow cells or specified cell populations in the femur were calculated and expressed as relative (percentage) to non-irradiated controls. To assess the effect of aspirin on irradiation-induced sickness, following total body irradiation, control and aspirin-treated mice were monitored daily for weight and. clinical severity. Clinical severity scoring was based on an arbitrary scale of 1 to 4, where 1 represented mice with mild but visible symptoms such as slowed activity and 4 represented those with severe morbidity, i.e., with lethargy, loose fecal pellet, piloerection, >20% weight loss, difficulty in breathing and movement and hence had to be euthanized. Experiments were done using adult mice (8–14 weeks old).

Antibodies and Reagents

Aspirin (Catalog# A2093), Doxorubicin (Catalog# D1515), DNA-PK inhibitor (DNA-PKi) Nu7026 (Catalog# N1537) and Histone acetyltransferase inhibitor (HATi) MG149 (Catalog# SML3011) were purchased from Sigma-Aldrich. The Ac-lysine antibody was from Santa Cruz (Catalog #sc-32268). Antibodies against H2A.X (Catalog #2595), γ-H2A.X (Catalog #2577), Histone H3 (Catalog #4499) and Histone H4 (Catalog #13919) were from Cell Signaling Technology. Alexa488-Anti-Sca-1(Catalog #11-5981-82) and Ac-H4K16 (Catalog # MA5-27794) were from Invitrogen and PECY7-Anti-cKit (Catalog #561681), Alexa Fluor® 700 Rat anti-Mouse CD45R (B220) (Catalog #557957) APC-Anti-CD11b (Catalog #553312), FITC-Anti-GR1 (Catalog #553126) were from BD Pharmingen. 2’,3’-cGAMP (Catalog #tlrl-nacga23), Pam3CSK4 (Catalog #tlrl-pms), Poly(I:C) (Catalog #tlrl-picwlv), Poly(dA:dT) (Catalog #tlrl-patn) were from InvivoGen. Ac-H4K16 (Catalog #ab109463) and Ac-H3K27 (Catalog #ab4729) antibodies were from Abcam.

Plasmids

pHPRT-DRGFP (# 26476) and pCBASceI (# 26477) [6], pimEJ5GFP (# 44026) [7] were obtained from Addgene. The mCherry-LacR-stop plasmid [8] was a gift from Nico Dantuma laboratory, Karolinska Institute, Stockholm, Sweden.

Microscopic visualization of chromatin compaction

The AO3 reporter cells [9] cultured in a 1:1 mixture of DME/Ham's F12 medium supplemented with antibiotics and 20% FCS to 70% density were transfected by lipofectamine with the 1 µg/ml mCherry-LacR-stop plasmid [8]. After 4 hours, they were treated with DMSO or indicated concentrations of aspirin (1 or 2 mM). 18 hours later samples were fixed with 4% paraformaldehyde and analyzed my fluorescence microscopy as described previously [10].

HR and NHEJ reporter assays

To assess the effect of aspirin on homologous recombination (HR) and NHEJ repair, briefly, the pHPRT-DRGFP (HR-reporter plasmid)[6]and the pimEJ5GFP (NHEJ reporter plasmid) [7] were stably transfected into HEK293T cells. 0.5×10^6 HEK293T stable reporter cells seeded in 6-well plates were transfected with 2 µg HA-I-SceI expression plasmid (pCBASce) then treated with aspirin or DMSO. 48 hours later, cells were analyzed by flow cytometry for GFP expression. Standard Mean of Error (±SEM) was calculated from three independent experiments.

Cells and cell culture

HEK293 cell and HEK293T cells were cultured under 5% CO2 at 37 °C in Dulbecco's modified Eagle medium (DMEM, high glucose, GlutaMAX) (Life Technologies) containing 10% (v/v) fetal bovin serum (FBS, GIBCO), 1% (v/v), penicillin (100 IU/ ml) + streptomycin (100 µg/ml). Bone-marrow- differentiating monocytes (BMDMos) were generated by culturing the mouse bone marrow cells in IMDM medium (GIBCO, Life Technologies) supplemented with 10% (v/v) FBS (GIBCO, Life Technologies), 1% (v/v) penicillin (100 U ml^{-1})/streptomycin (100 µg/ml), 2 mM glutamine (Sigma-Aldrich) and 10% (v/v) L929 conditional medium and maintained with 5% CO2 at 37 °C. The cells were used for experiment 4 days after start of differentiation. AO3 hamster cells, containing a 90-Mbp amplification of LacO sequences and flanking DNA[6], were cultured in a 1:1 mixture of DME/Ham's F12 medium supplemented with antibiotics and 20% FBS.

Generation of knockout cells

53BP1$^{-/-}$, *BRCA1*$^{-/-}$and non-target control (NTC) HEK293T cell lines were generated by CRISPR/Cas9 gene editing technology. Cells were transfected with the following gRNAs cloned into lentiCRISPR v2-puro: 53BP1 gRNA: CAGAATCATCCTCTAGAACC; NTC gRNA2: GTGTAGTTCGACCATTCGTG. BRCA1 gRNA1: TGCTAGTCTGGAGTTGATCA BRCA1 gRNA2: AAATCTTAGAGTGTCCCATC. Cells were selected with 10 mg mL-1 puromycin and resistant cells cloned.

Immunofluorescence

Cells were seeded and cultured on glass coverslips in 12 well plate and fixed in 4% paraformaldehyde (PFA) in PBS for 20 min at room temperature. Cells were permeabilized in 0.5% Triton X-100 for 10 min, blocked in 5% normal goat serum (NGS) then incubated with primary antibodies diluted in 1% NGS overnight at 4 °C, followed by incubation with indicated secondary antibodies diluted in 1% NGS at RT for 1 h then finally stained with DAPI for 15 min at room temperature. Coverslips were mounted using Dako Fluorescence Mounting Medium (Agilent) and imaged using Nikon confocal microscope (Eclipse C1 Plus). All scoring was performed under blinded conditions. γH2A.X, BRCA1, and 53BP1 foci were counted from 40 microscopic fields containing approx. 300 cells from 3 independent experiments.

Chromatin fractionation and immunoblotting

To isolate the chromatin, we used the Subcellular Protein Fractionation Kit (Thermo Fisher) according to the manufacturer's instructions and as previously described[11]. Proteins were quantified by BCA reagent (Thermo Fisher Scientific, Rockford, IL). Samples were resolved in SDS-PAGE, transferred to nitrocellulose membrane (Amersham Protran 0.45 μm NC) and immunoblotted with specific primary antibodies followed by HRP-conjugated secondary antibodies. Protein bands were detected by Supersignal West Pico or Femto Chemiluminescence kit (Thermo Fisher Scientific).

Alternatively, cells were lyzed in mild Nonidet P-40 lysis buffer (1% NP-40, 50 mM Tris–HCl, 150 mM NaCl, pH 7.5,1 mM NaF, 2 mM PMSF, protease inhibitor cocktail [Roche AppliedScience], 1 mM sodium orthovanadate, and 10 mM sodiumpyrophosphate). After centrifugation at 10,000g for 15 min at 4°C, proteins in supernatants were quantified by BCA reagent (Thermo Fisher Scientific, Rockford, IL). Proteins were resolved in SDS–PAGE, transferred to nitrocellulose membrane (Amersham Protran 0.45 μm NC) and immunoblotted with specific primary antibodies. Protein bands were detected by SuperSignal West Pico or FemtoChemiluminescence Kit (Thermo Fisher Scientific).

Inflammasome activation analysis

Analysis of inflammasome activation was done as previously described [12-14]. Briefly, BMDMos seeded in the density of 1.5 x 10^6 cells/well were treated with aspirin overnight and then primed with 500 ng/ml LPS for 4 h. Cells were then transfected with 1 μg/ml poly(dA:dT) for 1 h using Lipofectamine 2000 (Invitrogen). Supernatants were collected. Proteins were precipitated using chloroform:methanol extraction and re-suspended in 2 x Laemmli buffer. Cells were lysed in 2 x Leammli buffer. Samples were separated on 13.5% SDS-PAGE gel and analysed for activation of Caspase-1 and IL-1β by immunoblotting, as described in the section above.

In vitro protein acetylation assay by aspirin

Chromatins fractions isolated as described above were incubated with indicated concentration of aspirin in reaction buffer (40 mM Tris–HCl, 5 mM MgCl2, 100 mM NaCl) for 1 hour at 37 °C. The mixture was boiled in loading buffer and analyzed by immunoblotting.

Analysis of DNA repair by Comet assay

Cells were subjected to the indicated doses of γ-irradiation or doxorubicin and chromosome fragmentation was determined by comet assay as previously described [10,11,15]. Briefly, during irradiation cells were kept on ice to stop the DNA repair process. Thereafter, cells were transferred to 37°C to allow DNA repair to occur for indicated duration. Cells were then harvested by brief centrifugation and resuspension in cold PBS. Cells were mixed with 1% low-melting agarose (40°C) at a ratio of 1:3 vol/vol) before pipetting onto CometSlides. Slides were then immersed in prechilled lysis buffer (1.2 M NaCl, 100 mM EDTA, 0.1% sodium lauryl sarcosinate, 0.26M NaOH PH>13) for overnight (18-20 h) lysis at 4°C in the dark. Slides were carefully removed and submerged in room temperature rinse buffer (0.03 M NaOH and 2 mM EDTA, pH > 12) for 20 min in the dark. This washing step was done 2 times. Slides were transferred to a horizontal electrophoresis chamber containing rinse buffer and separated for 25 min at voltage (0.6 V/cm). Finally, slides were washed with distilled water and stained with 10 µg/ml propidium iodide and analyzed by fluorescence microscopy. 20 fields with about 200 cells in each sample were evaluated and quantified by the Fiji software to determine the tail length (tail moment).

Determination of micronuclei

HEK293 cells pre-treated with aspirin (1 mM) then exposed to γ-irradiation (or not) were cultured for 24 hours, then fixed (4% PFA), permeabilized (0.5% Triton X- 100), DAPI stained then analyzed by microscopy as described previously [10,11,15]. Micronuclei were defined as discrete DNA aggregates separate from the primary nucleus in cells where interphase primary nuclear morphology was normal. Cells with an apoptotic or necrotic appearance were excluded.

RT-qPCR

Total RNA was extracted using the Trizol (Thermo Fisher) according to the manufacturer's protocol. cDNA was prepared using Maxima H Minus First Strand cDNA Synthesis Kit and random oligomer primers (Thermo Fisher Scientific). Real-time qPCR was performed by using QuantStudio 5. The results were normalized to 18s (reference gene) and expressed as fold change relative to untreated or mock-treated controls using the comparative CT method (ΔΔCT). The following TaqMan Gene Expression Assays (FAM) (Applied Biosystems, Thermo Fisher Scientific) in combination with the TaqMan Gene Expression Master Mix (#4369016; Applied Biosystems, Thermo Fisher Scientific) were applied: Ifnβ (Mm00439552_s1), Mx1 (Mm00487796_m1), Tnfα (Mm00443258_m1) and Rn18s (Mm03928990_g1).

Statistical Analysis

Statistical analysis was performed by GraphPad Prism 5.0 software. All of the data shown in the histograms were the results of at least three independent experiments and are presented as the mean ± SEM or mean ± SD. The sample size (n) for each statistical analysis and statistical methods used to assess significant differences are indicated in figure legends. Differences between values were considered statistically significant when $*P < 0.05$, $**P < 0.01$, $***P < 0.001$, and $****P < 0.0001$.

Supplementary References

1 Sauer, J. D. *et al.* The N-ethyl-N-nitrosourea-induced Goldenticket mouse mutant reveals an essential function of Sting in the in vivo interferon response to Listeria monocytogenes and cyclic dinucleotides. *Infect Immun* **79**, 688-694, doi:10.1128/IAI.00999-10 (2011).
2 Adachi, O. *et al.* Targeted disruption of the MyD88 gene results in loss of IL-1- and IL-18-mediated function. *Immunity* **9**, 143-150, doi:10.1016/s1074-7613(00)80596-8 (1998).
3 Kumar, H. *et al.* Essential role of IPS-1 in innate immune responses against RNA viruses. *J Exp Med* **203**, 1795-1803, doi:10.1084/jem.20060792 (2006).
4 Hartlova, A. *et al.* DNA damage primes the type I interferon system via the cytosolic DNA sensor STING to promote anti-microbial innate immunity. *Immunity* **42**, 332-343, doi:10.1016/j.immuni.2015.01.012 (2015).
5 Erttmann, S. F. *et al.* The gut microbiota prime systemic antiviral immunity via the cGAS-STING-IFN-I axis. *Immunity* **55**, 847-861 e810, doi:10.1016/j.immuni.2022.04.006 (2022).
6 Pierce, A. J., Hu, P., Han, M., Ellis, N. & Jasin, M. Ku DNA end-binding protein modulates homologous repair of double-strand breaks in mammalian cells. *Genes Dev* **15**, 3237-3242, doi:10.1101/gad.946401 (2001).
7 Bennardo, N., Cheng, A., Huang, N. & Stark, J. M. Alternative-NHEJ is a mechanistically distinct pathway of mammalian chromosome break repair. *PLoS Genet* **4**, e1000110, doi:10.1371/journal.pgen.1000110 (2008).
8 Luijsterburg, M. S. *et al.* A new non-catalytic role for ubiquitin ligase RNF8 in unfolding higher-order chromatin structure. *EMBO J* **31**, 2511-2527, doi:10.1038/emboj.2012.104 (2012).
9 Tumbar, T., Sudlow, G. & Belmont, A. S. Large-scale chromatin unfolding and remodeling induced by VP16 acidic activation domain. *J Cell Biol* **145**, 1341-1354, doi:10.1083/jcb.145.7.1341 (1999).
10 Jiang, H., Swacha, P. & Gekara, N. O. Nuclear AIM2-Like Receptors Drive Genotoxic Tissue Injury by Inhibiting DNA Repair. *Adv Sci (Weinh)* **8**, e2102534, doi:10.1002/advs.202102534 (2021).
11 Jiang, H., Panda, S. & Gekara, N. O. Comet and micronucleus assays for analyzing DNA damage and genome integrity. *Methods Enzymol* **625**, 299-307, doi:10.1016/bs.mie.2019.05.015 (2019).
12 Erttmann, S. F. *et al.* Loss of the DNA Damage Repair Kinase ATM Impairs Inflammasome-Dependent Anti-Bacterial Innate Immunity. *Immunity* **45**, 106-118, doi:10.1016/j.immuni.2016.06.018 (2016).
13 Swacha, P., Gekara, N. O. & Erttmann, S. F. Biochemical and microscopic analysis of inflammasome complex formation. *Methods Enzymol* **625**, 287-298, doi:10.1016/bs.mie.2019.05.014 (2019).
14 Erttmann, S. F. & Gekara, N. O. Hydrogen peroxide release by bacteria suppresses inflammasome-dependent innate immunity. *Nat Commun* **10**, 3493, doi:10.1038/s41467-019-11169-x (2019).
15 Jiang, H. *et al.* Chromatin-bound cGAS is an inhibitor of DNA repair and hence accelerates genome destabilization and cell death. *EMBO J* **38**, e102718, doi:10.15252/embj.2019102718 (2019).

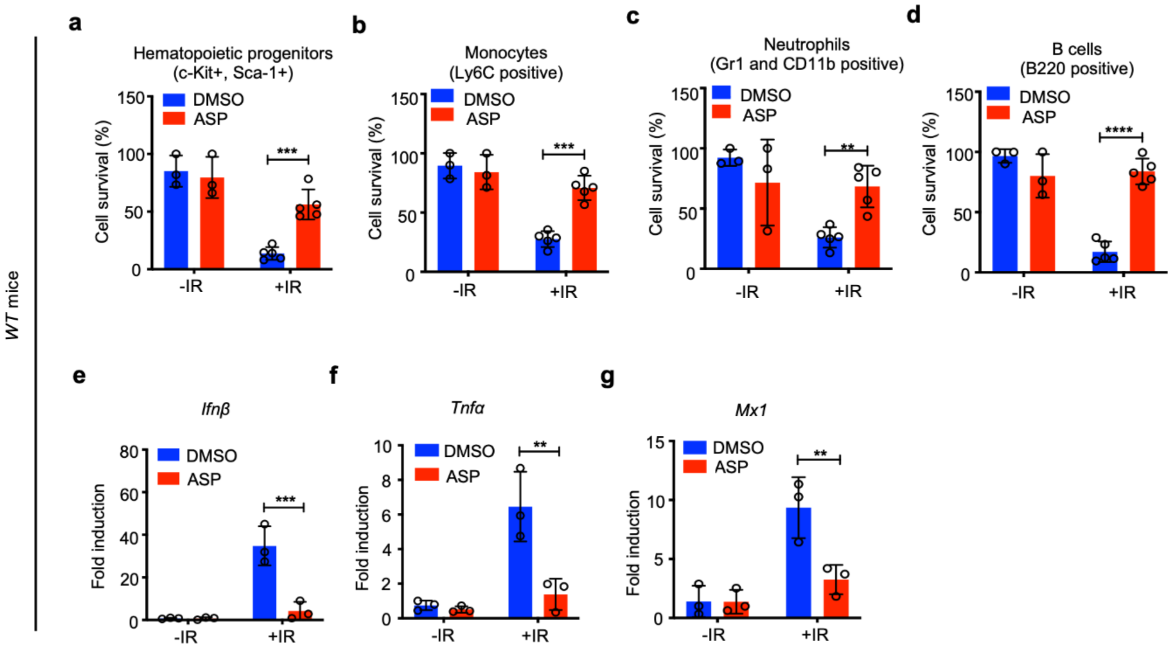

Supplementary information, Fig. S1. Aspirin protects against irradiation-induced bone marrow injury in WT mice. a-d Survival of the hematopoietic progenitors (**a**) monocytes (**b**), neutrophils (**c**) and B cells (**d**) in bone marrow 10 hours post irradiation (IR: 9 Gy) of WT mice pretreated with aspirin (n=8) or DMSO (n=8). **e-g** Transcripts of *Ifnβ* (**e**), *Tnfα* (**f**), and *Mx1* (**g**) in bone marrow cells isolated from aspirin or DMSO pre-treated WT mice, 10 hours post irradiation (IR: 9 Gy). The data are are presented as mean ± SD. Statistical significance is assessed using one-way ANOVA followed by Tukey's multiple comparisons test.

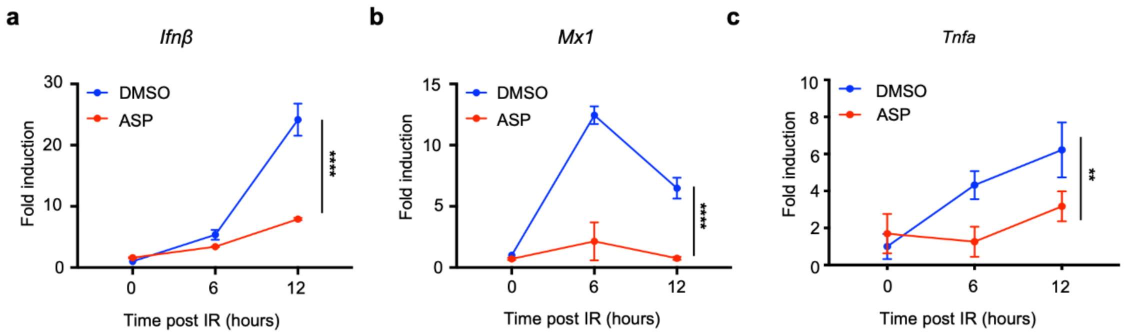

Supplementary information, Fig. S2. Aspirin inhibits irradiation-induced inflammatory gene expression. a-c Bone marrow derived monocytes (BMDMos) were pre-treated with aspirin or DMSO for 12 hours then analyzed for the transcripts of the indicated inflammatory mediators; *Ifnβ* (**a**), *Mx1* (**b**) and *Tnfα* (**c**) at indicated time points after irradiation (9 Gy). The data are presented as mean ± SD. Statistical significance are assessed using two-way ANOVA test.

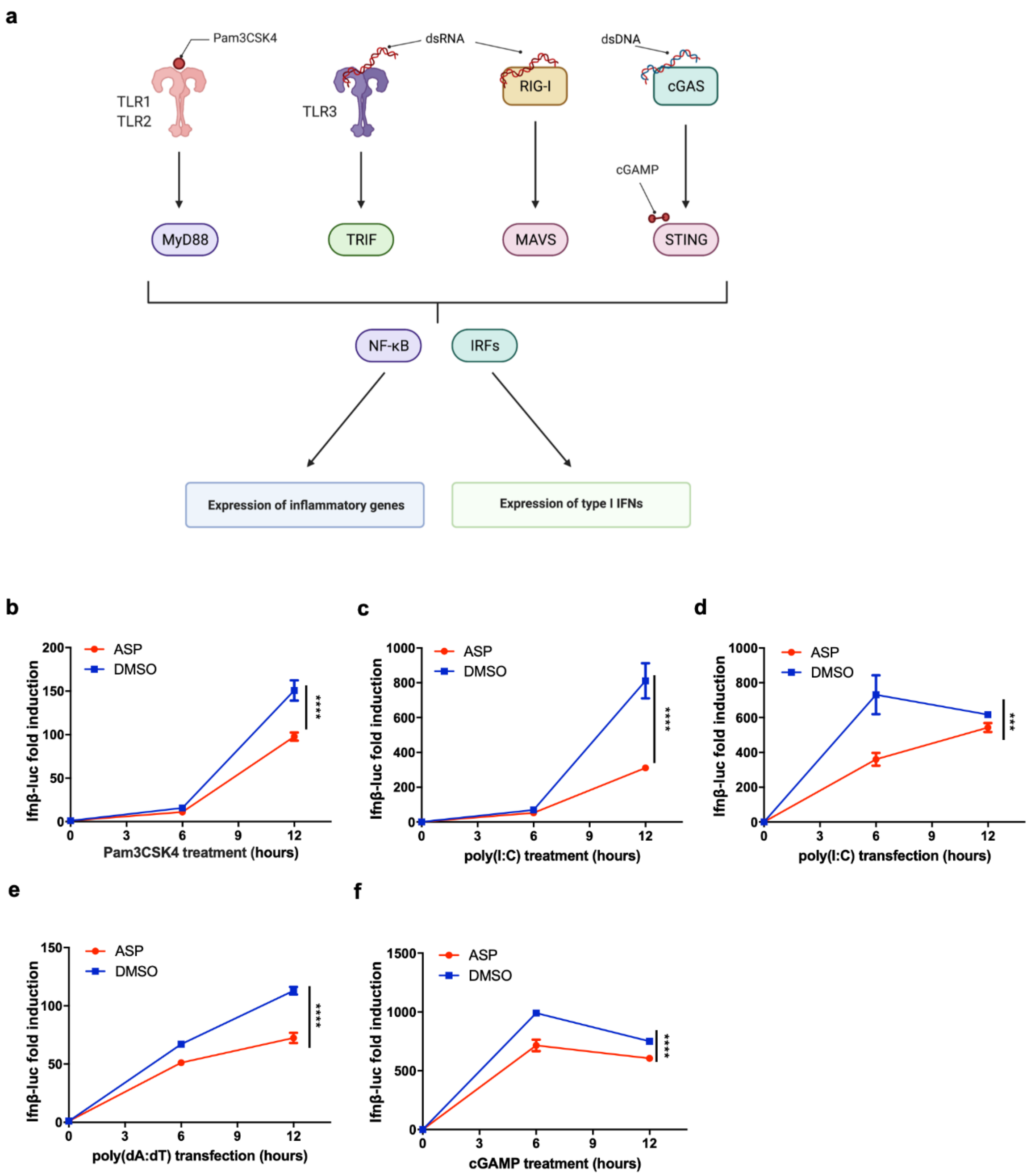

Supplementary information, Fig. S3. Aspirin inhibits multiple PRR pathways for inflammatory gene induction. a Schematic overview of PRR pathways for inflammatory gene activation. **b-f,** *Ifnβ* induction in *Ifnb*$^{+/\Delta\beta\text{-}luc}$ BMDMos that were pre-treated with DMSO or aspirin (2 mM) then stimulated (or not) with 1 µg/ml Pam3CSK4 (**b**), 10 µg/ml poly(I:C) (**c**), 2 µg/ml poly(I:C) transfection (**d**), 1 µg/ml poly(dA:dT) transfection (**e**) 1 µg/ml cGAMP (**f**) then analyzed for luciferase activity. The data are presented as mean ± SD, n=3. Statistical significance are assessed using two-way ANOVA test.

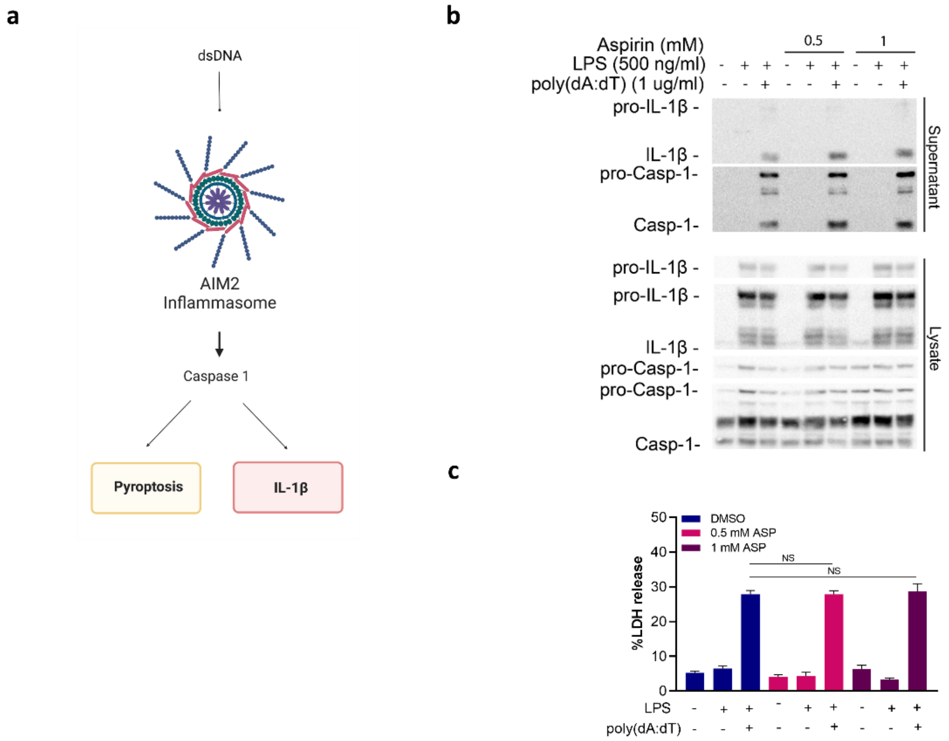

Supplementary information, Fig. S4. Effect of aspirin on AIM2 inflammasome. a Schematic summary of AIM2 inflammasome mediated pyroptosis and IL-1β secretion. **b** Immunoblot analysis of indicated proteins in supernatants or lysates of BMDMos that were pre-treated with aspirin (0.5 or 1 mM) then primed with LPS and transfected with the AIM2 agonist poly(dA:dT). **c** Estimation of pyroptosis by LDH release in BMDMos pretreated with aspirin then transfected with poly(dA:dT). The data are presented as mean ± SD, n=3. Statistical significance are assessed using one-way ANOVA test. NS P>0.05

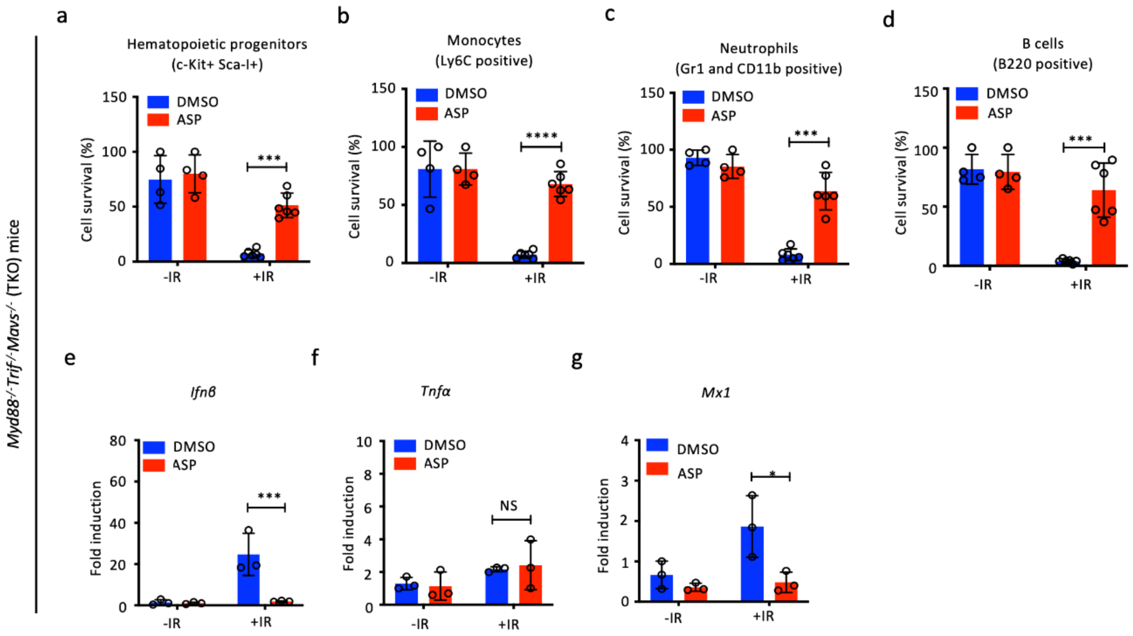

Supplementary information, Fig. S5. Aspirin protects against irradiation-induced bone marrow injury in TKO mice. a-d Survival of the hematopoietic progenitors (**a**) monocytes (**b**), neutrophils (**c**) and B cells (**d**) in bone marrow 10 hours post-irradiation (IR: 9 Gy) of TKO mice pretreated with aspirin (n=10) or DMSO (n=10). **e-g** Transcripts of *Ifnβ* (**e**), *Tnfα* (**f**), and *Mx1*(**g**) in bone marrow cells isolated from aspirin or DMSO pre-treated TKO mice, 10 hours post irradiation (IR: 9 Gy). The data are presented as mean ± SD. Statistical significance is assessed using one-way ANOVA followed by Tukey's multiple comparisons test.

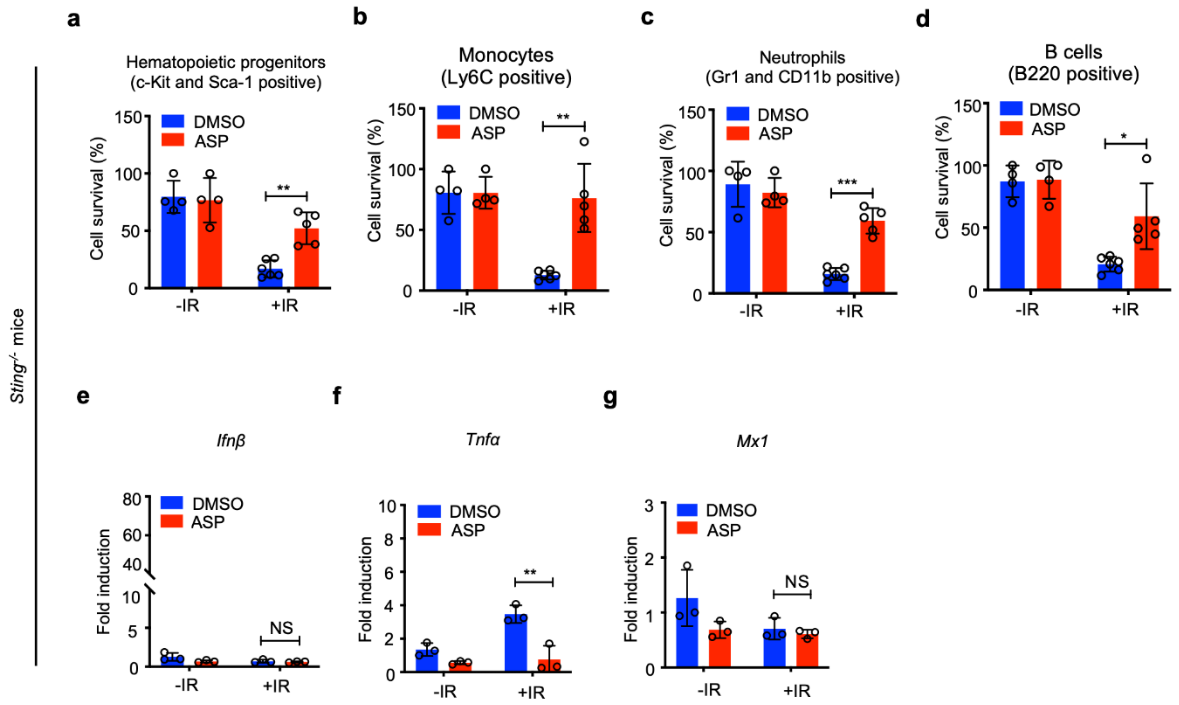

Supplementary information, Fig. S6. Aspirin protects against irradiation-induced bone marrow injury in *Sting*$^{-/-}$ mice. a-d Survival of the hematopoietic progenitors (**a**) monocytes (**b**), neutrophils (**c**) and B cells (**d**) in bone marrow 10 hours post irradiation (IR: 9 Gy) of in *Sting*$^{-/-}$ mice pretreated with aspirin (n=10) or DMSO (n=10). **e-g** Transcripts of *Ifnβ* (**e**), *Tnfα* (**f**), and *Mx1*(**g**) in bone marrow cells isolated from aspirin or DMSO pre-treated *Sting*$^{-/-}$ mice, 10 hours post irradiation (IR: 9 Gy). The data are presented as mean ± SD. Statistical significance is assessed using one-way ANOVA followed by Tukey's multiple comparisons test.

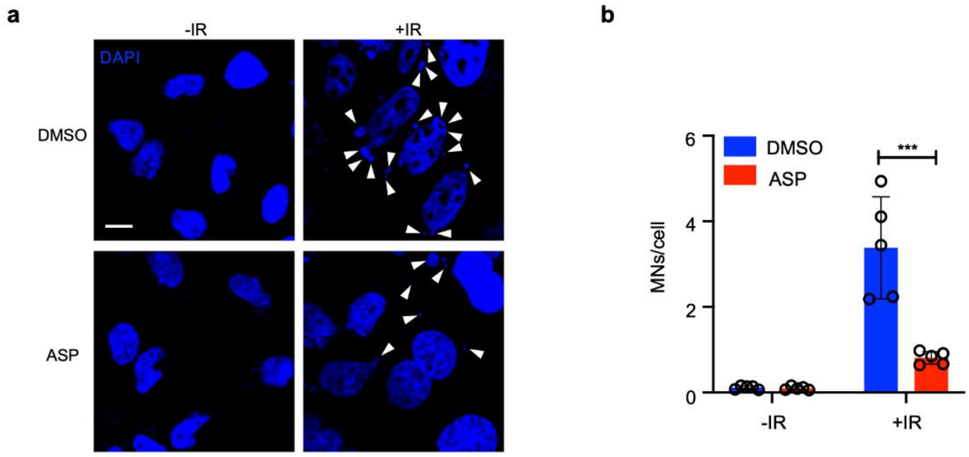

Supplementary information, Fig. S7. Aspirin inhibits DNA damage-induced micronuclei generation. a Confocal microscopic visualization of micronuclei (indicated by arrowhead) in HEK293 cells pre-treated with DMSO or aspirin before exposure to γ-irradiation (9 Gy). Scale bar=10 μm. **b** Average MNs/cell in corresponding representative images. Bar graphs show mean values from five different microscopic fields with over 200 cells. Graphs show as mean ±SEM and statistical significance were assessed using One-way ANOVA followed by Tukey's multiple comparisons test. ***$P < 0.001$.

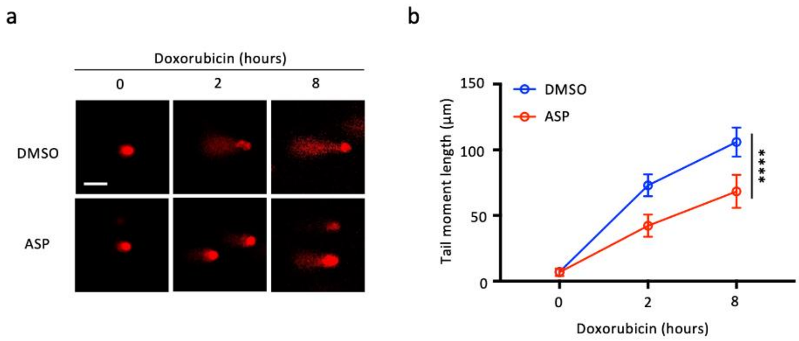

Supplementary information, Fig. S8. Aspirin protect against chemotherapy-induced DNA damage. a Comet tails in BMDMos pre-treated (or not) with Aspirin (1 mM) for 4 hours then stimulated with doxorubicin (1 μM) for indicated duration. **b** Corresponding quantification of the comet tail moments from 20 different fields with n > 200 comets of three independent experiments. Scale bar=50 μm._The data presented as mean ± SEM, n = 200. ∗∗∗P< 0.001, ∗∗∗∗P< 0.0001, NS P>0.05 (one-way ANOVA test).

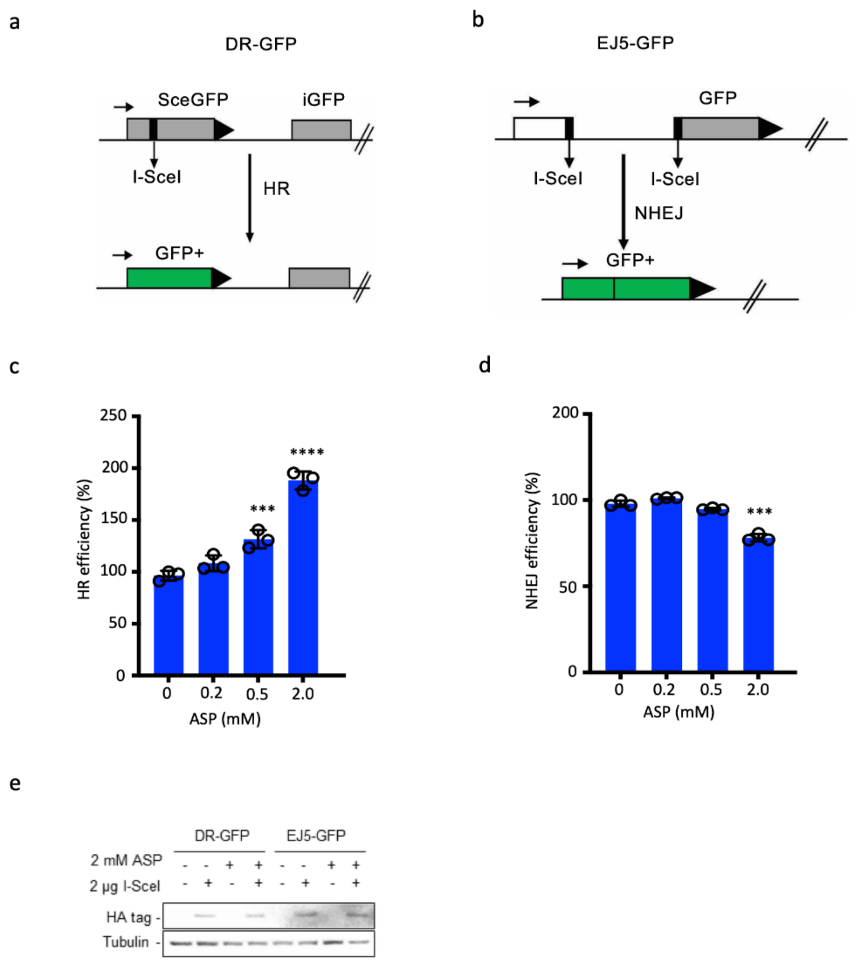

Supplementary information, Fig. S9. Aspirin promotes HR-DNA repair. a, b Schematics of (**a**) HR-GFP and (**b**) NHEJ-GFP reporter assays. **c, d** DNA repair efficiency in HR-GFP reporter (**c**) or NHEJ-GFP reporter cells (**d**) pretreated with DMSO or indicated concentrations of aspirin. (**e**) Expression levels of HA-I-SceI (the DNA break-inducing nuclease) in corresponding samples in c, d that were pre-treated with (or not) 2 mM aspirin.

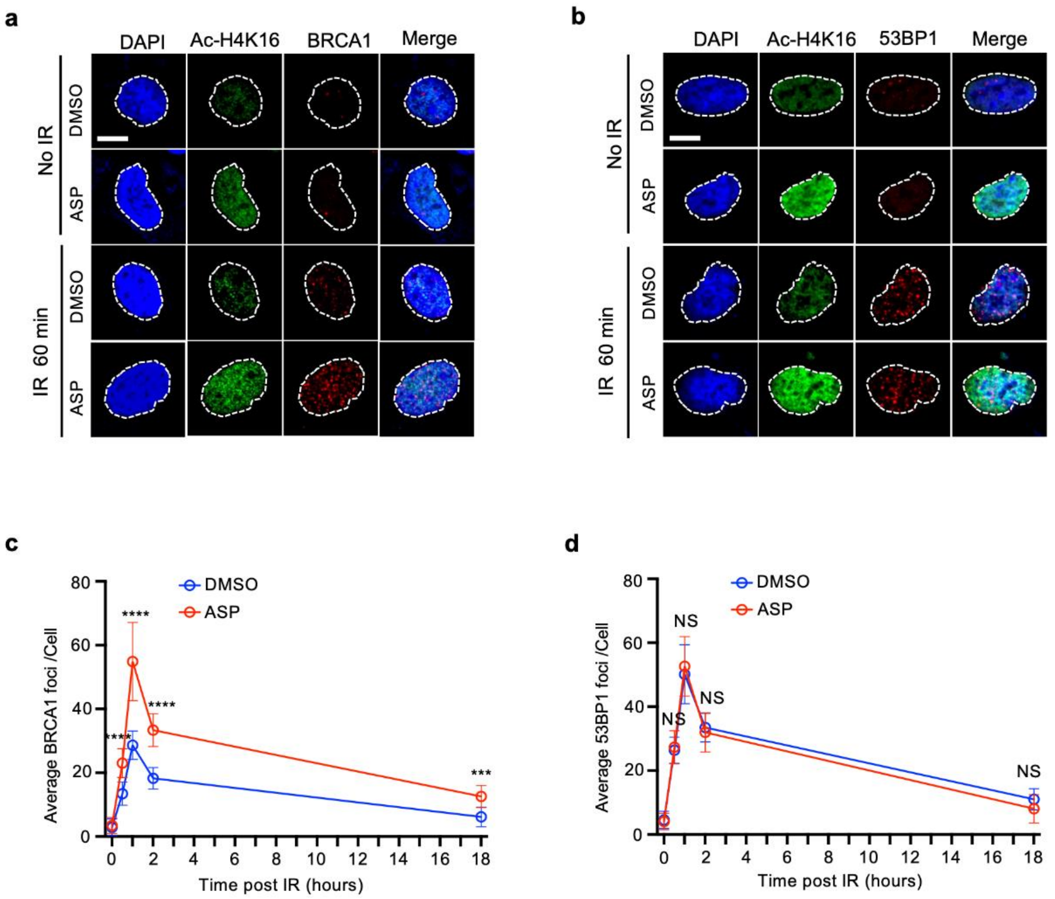

Supplementary information, Fig. S10. Aspirin acetylates H4K16 and promotes the recruitment of BRCA1 but not 53BP1 to DNA damage sites. a Immunofluorescence images of BRCA1 and Ac-H4K16 1 hour after irradiation (9 Gy) of HEK293 cells pre-treated with DMSO or aspirin. Scale bar = 10 μm. **b** Immunofluorescence images of 53BP1 and Ac-H4K16 1 hour after irradiation (9 Gy) of HEK293 cells pre-treated with DMSO or aspirin. Scale bar = 10 μm. **c, d** Time course quantification of BRCA1(**c**) and 53BP1(**d**) foci per nucleus in HEK293 cells pretreated (or not) with aspirin. Graphs were shown as mean ± SEM, n = 40. ∗∗∗P< 0.001, ∗∗∗∗P< 0.0001, NS P>0.05 (one-way ANOVA test).

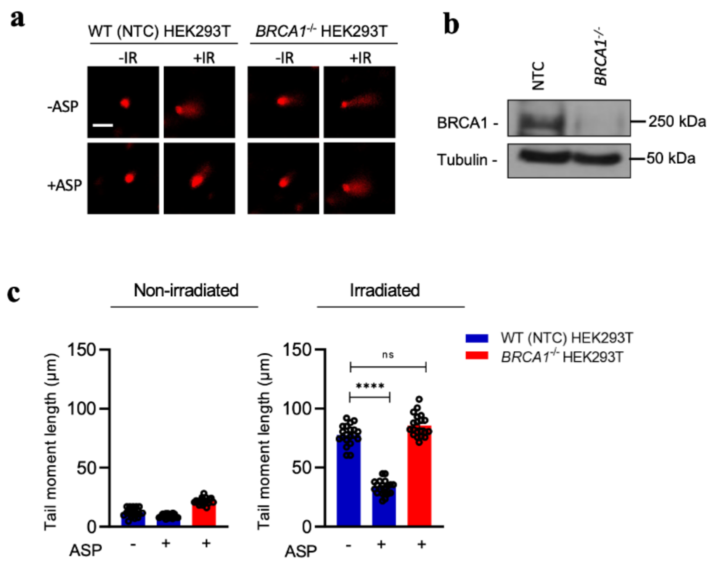

Supplementary information, Fig. S11. Potentiation of DNA repair by aspirin requires BRCA1. a Representative comet tails of Non-Target Control (NTC) or *BRCA1*$^{-/-}$ HEK293T that were pre-treated (or not) with aspirin (2 mM), then irradiated (9 Gy) on ice, followed by incubation at 37°C to allow DNA repair to occur for the indicated duration. Scale bar=50 µm. **b** Immunoblot analysis of BRCA1 expression and Tubulin (control) in NTC and *BRCA1*$^{-/-}$ HEK293T cells. **c** Quantification of the comet tail moments from 20 different fields with n > 200 comets of three independent experiments. Graphs were shown as mean ± SEM, n = 200. ∗∗∗∗P< 0.0001, ns P>0.05 (one-way ANOVA test).

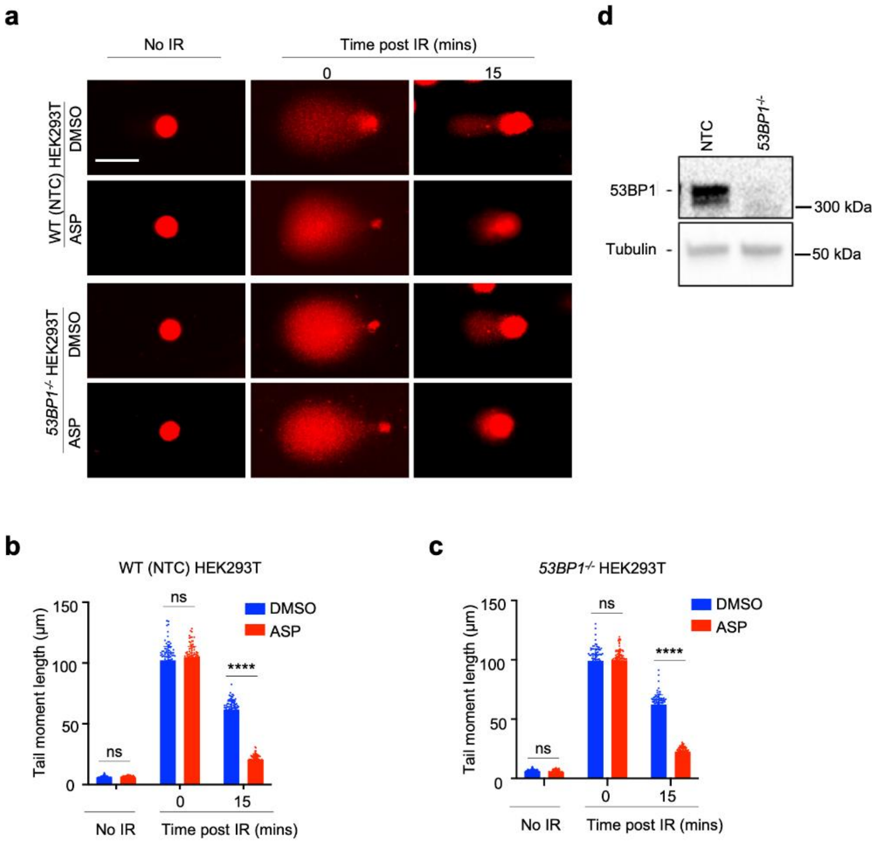

Supplementary information, Fig. S12. Aspirin promotes DNA repair independently of the NHEJ checkpoint protein 53BP1. a Representative comet tails of Non-Target Control (NTC) or *53BP1*$^{-/-}$ HEK293T that were pre-treated (or not) with aspirin (2 mM), then irradiated (9 Gy) on ice, followed by incubation at 37°C to allow DNA repair to occur for the indicated duration. Scale bar=50 µm. **b, c** Corresponding quantification of the comet tail moments from 20 different fields with n > 200 comets of three independent experiments. Graphs were shown as mean ± SEM, n = 200. ∗∗∗∗P< 0.0001, ns P>0.05 (one-way ANOVA test). **d,** 53BP1 and Tubulin (control) levels in NTC and *53BP1*$^{-/-}$ HEK293T cells.

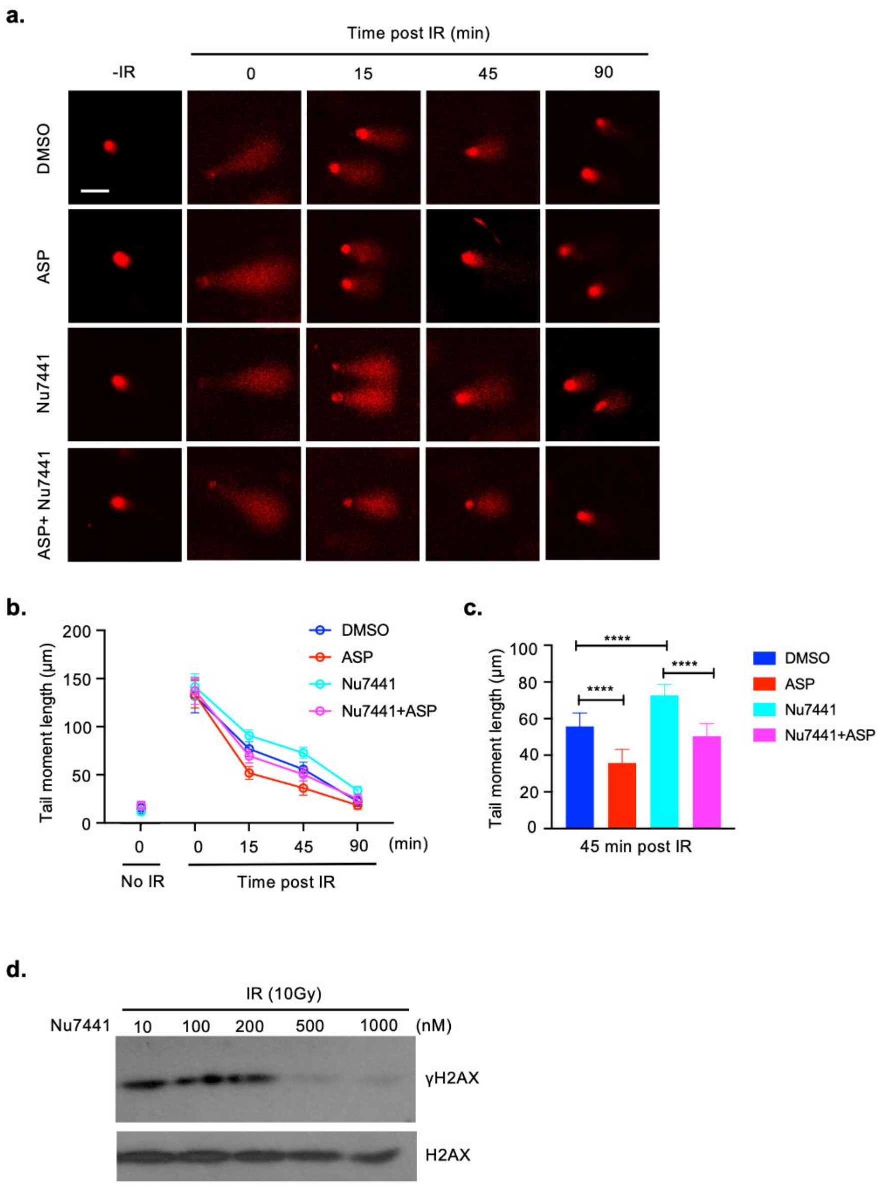

Supplementary information, Fig. S13. Aspirin promotes DNA repair independently of the NHEJ kinase DNA-PKC. a Representative comet tails of BMDMos pre-treated (or not) with aspirin (2 mM) and DNA-PK inhibitor (DNA-PKi) Nu7026 (500 nM) alone or in combination, then irradiated (9 Gy) on ice, followed by incubation at 37°C to allow DNA repair for indicated duration. Scale bar=50 μm. **b**, **c** Corresponding quantification of the comet tail moments from 20 different fields with n > 200 comets of three independent experiments. (**b**) Kinetic graph of DNA repair. (**c**) Comparison of the DNA damage level in the indicated treatment groups 45 minutes after radiation. **d** Titrated effect of DNA-PK inhibitor (DNA-PKi) Nu7026 on DNA damage-induced γH2AX. H2A.X was used as loading control. Graphs were shown as mean ± SEM, n = 200. ∗∗∗P< 0.001, ∗∗∗∗P< 0.0001, NS P>0.05 (one-way ANOVA test).

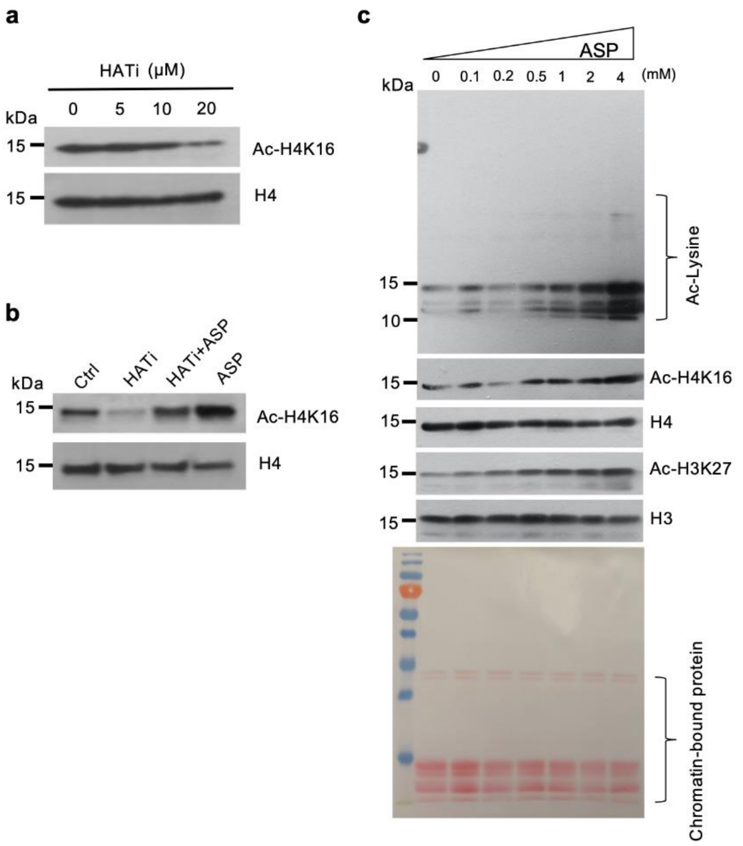

Supplementary information, Fig. S14. Aspirin acetylates H4-K16 directly by its acetyl group. a Aspirin acetylates H4K16 independently of Histone acetyl transferases. Immunoblot of Ac-H4K16 and total H4 in BMDMos treated for 6 hours with indicated concentrations of the histone acetyltransferase inhibitor (HATi) MG149. **b** Ac-H4K16 and total H4 in BMDMos treated with aspirin (2 mM), HATi (20 μM) or both for 6 hours. **c** Aspirin directly donates acetyl group to H4K16. Chromatin fractions isolated from BMDMos pre-incubated with indicated concentrations of aspirin at 37 °C for 1 hour then immunoblotted for Ac-H4K16 and total acetylated proteins.

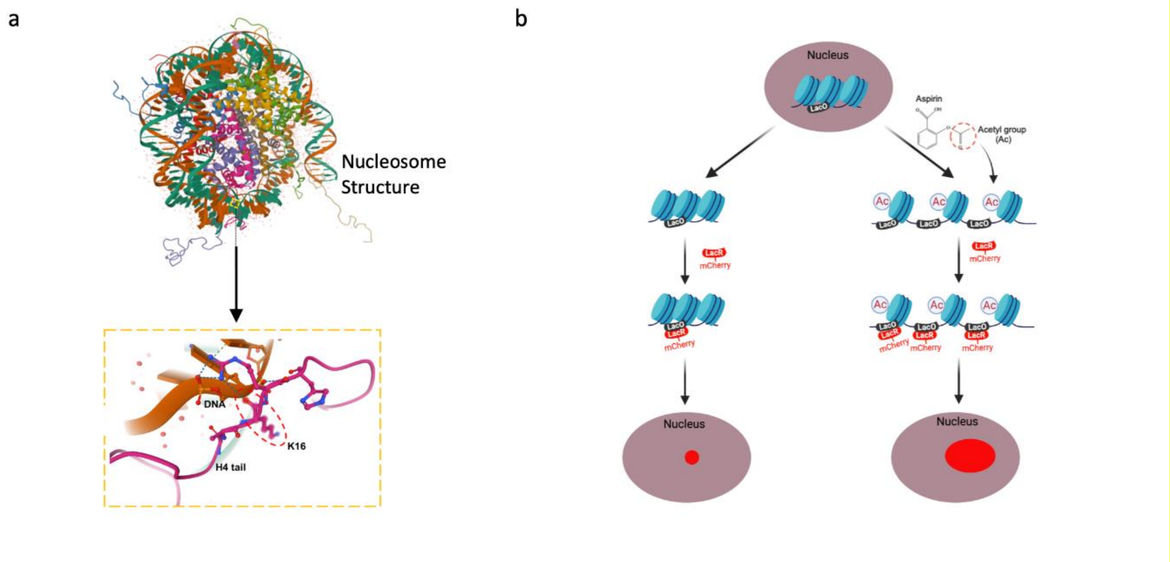

Supplementary information, Fig. S15. Aspirin promotes H4-K16 acetylation and DNA damage-associated chromatin decompaction. a Overview of a single nucleosome complex with a depiction of the histone H4-dsDNA interface and histone H4K16 location. **b** Schematic overview of the chromatin compaction assay and effect of aspirin on chromatin.
